# Defining successful treatment of neurogenic orthostatic hypotension with droxidopa in a patient with multiple system atrophy

**DOI:** 10.1007/s10286-017-0432-8

**Published:** 2017-06-19

**Authors:** Brent P. Goodman, Fiona Gupta

**Affiliations:** 1 Department of Neurology, Mayo Clinic Arizona, Scottsdale, AZ USA; 2 Movement Disorders Center, Hackensack University Medical Center, Hackensack, NJ USA

**Keywords:** Multiple system atrophy, Neurogenic orthostatic hypotension

## Challenge question

When using droxidopa, what are the criteria to adjust the dosage, combine it with other medications, and define therapeutic success?

## Case presentation

Mrs. M is an 80-year-old woman with the parkinsonian variant of multiple system atrophy (MSA-P). She initially presented with a 3-year progressive history of imbalance and a slow gait. She reported frequent falls, a decline in the ability to complete activities of daily living (ADLs), and noted some uncontrollable jerkiness of her arms (not tremors). She later developed facial dyskinesia shortly after starting treatment with levodopa. She has been ambulatory with a walker and lives in an assisted living facility. Her past medical history is significant for hyperlipidemia, urinary urgency, occasional incontinence, and constipation. Her medications included atorvastatin 10 mg taken orally once daily (QD), oxybutynin 5 mg twice daily (BID), and docusate sodium 100 mg BID.

Approximately 6 months ago, Mrs. M had several episodes of syncope, all occurring when she was rising from a seated to a standing position or when getting out of bed. At that time, orthostatic blood pressure (BP) readings confirmed that she was experiencing a large fall in BP (170/87 mmHg supine to 110/65 mmHg after 3-min of standing-up) while her heart rate (HR) increased by only 10 beats per minute or less; therefore, she was diagnosed with neurogenic orthostatic hypotension (nOH). Droxidopa was initiated at 100 mg on a modified three times daily (TID) schedule (taken on awakening, at midday, and at least 3–4 h prior to bedtime) [1, 3]. Her droxidopa dose was up-titrated every 24–48 h by 100 mg on the modified TID schedule. She had a good symptomatic response with droxidopa at 500 mg on the modified TID schedule and was experiencing much less frequent episodes of dizziness and lightheadedness, and no syncope.

## Expert commentary (Dr. Goodman)

Prior to the initiation of droxidopa for nOH in this patient with MSA-P, it is important to identify goals of treatment and, ideally, to discuss these treatment goals with patients and their caregivers, when relevant. In patients with MSA, symptoms of nOH may include syncope, near syncope, lightheadedness when standing, fatigue, headache, or confusion. Recognition of these various symptoms provides not only the rationale for pursuing pharmacotherapy with droxidopa, but also will help establish treatment goals for individual patients.

## Case continuation

The patient was on a stable dose of droxidopa 500 mg on the modified TID schedule for approximately 6 months. However, she presented again with recurrent syncope, as well as other symptoms of nOH in a follow-up visit.

## Expert commentary (Dr. Goodman)

Syncope is a common manifestation of nOH in patients with MSA, and reducing the frequency of syncopal episodes is a major goal of treatment with droxidopa. A reduction in syncope in this patient for the initial 6 months following droxidopa initiation could be considered a treatment success. It is not uncommon for patients with nOH to have treatment reduce, but not fully abolish, nOH symptoms. Patients may also experience an exacerbation in symptoms during periods of prolonged bed rest or concomitant illness, including a recurrence of syncope. When evaluating a patient with worsening symptoms, including recurrent syncopal episodes, it is critical to rule out new medical problems (such as anemia or cardiac arrhythmia) or new medication that has resulted in new or recurrent symptoms. As such, a review of the patient’s interval medical history and medications should be conducted. In addition, basic blood work and an electrocardiogram should also be ordered.

## Case continuation

Review of medications, basic blood work, and electrocardiogram showed that were no new factors aggravating the fall in BP when standing. Thus, it was concluded that the likely cause of her recurrent syncope was progressing neurodegeneration, affecting autonomic pathways involved in BP control.

In this situation, for both patients and their caregivers, it is important to review again the non-pharmacologic measures to increase BP, including liberalization of salt and fluid intake, and wearing compression garments such as compression stockings or an abdominal binder. It is not uncommon for patients with MSA to forget some or all of these critically important lifestyle measures that are useful in enhancing the effectiveness of droxidopa. The patient and caregivers at the assisted living facility were instructed to make use of several of these lifestyle modifications; they were also instructed to ensure that the patient rested or slept with the head of the bed elevated by at least 30° in order to avoid supine hypertension, a common complication in patients with MSA [2].

After reviewing again for potential medical and medication causes for new or worsening symptoms, adjustments in the patient’s pharmacotherapeutic regimen should be considered. A mistake often made in situations such as this is to assume that droxidopa or other medications have failed and should be discontinued or substituted with an alternative medication.

In this patient with MSA-P, the cessation of syncope for 6 months after starting droxidopa indicated a significant treatment success, but she remained symptomatic. As the patient was not at the maximal dose of droxidopa, it was decided to up-titrate the patient to a higher dose of droxidopa, with the treatment goal of reducing or eliminating syncope. The patient was titrated up to the maximum dose of droxidopa at 600 mg on the modified TID schedule. Unfortunately, even at the highest dosage of droxidopa, this patient continued to experience episodes of syncope. Her supine BP was 167/107 mmHg with a HR of 71 bpm, which decreased to 95/59 mmHg with a HR of 83 bpm upon standing for 3 min. In some circumstances, such as with this patient with MSA-P, despite maximal droxidopa doses, it may be necessary to add other medications for orthostatic hypotension.

Midodrine [6] is effective to treat nOH and syncope, starting at low dosages, and titrating upward with close monitoring. Sustained hypertension may be the limiting factor when combining droxidopa and midodrine, although no dedicated study has been specifically performed. Because syncope and other symptoms of nOH are typically worse in the mornings, higher doses of these medications might be used in the mornings, with smaller doses, or no adjunctive medications at all, administered in the afternoon or evening. For this patient, midodrine at 5 mg on a modified BID schedule (taken on awakening and at midday) was added to her droxidopa regimen at 600 mg TID. She was then titrated up to 10 mg of midodrine on the modified BID schedule. While titrating midodrine, seated BP should be checked 1 h after each midodrine dose to ensure that the pressor effect is not too high. After reaching a steady dose of midodrine, she obtained adequate symptom control and was experiencing significantly fewer episodes of syncope, pre-syncope, dizziness, and lightheadedness. Her supine BP was 174/108 mmHg with a HR of 68 bpm, which decreased to 110/89 mmHg with a HR of 79 bpm upon standing for 3 min.

In addition to droxidopa and midodrine, other off-label medications could be considered in patients with MSA who are experiencing severe symptomatic nOH. Pyridostigmine [5] is a medication that has been used in nOH. It may be used in combination with droxidopa, although no dedicated study has been specifically performed. Desmopressin can be administered to patients with frequent nocturnal enuresis, which is common in MSA, and may help to decrease symptoms of nOH and syncope in the morning in these patients, although this has been shown in open-label small series (<10 patients) [4]. However, hyponatremia is a dangerous side effect of desmopressin.

## Expert commentary (Dr. Gupta)

MSA-P is one of the most challenging neurodegenerative disorders to treat. In addition to severe autonomic dysfunction, there are multiple motor and non-motor symptoms that need to be addressed. Levodopa can be used, typically with only minimal benefit, and may worsen nOH. The degree of nOH in these patients is typically quite severe, and often a major cause of disability. Non-pharmacologic therapies are often ineffective alone; therefore, consideration of pharmacologic therapies—whether monotherapy or adjunctive therapy—is paramount. In this patient, droxidopa was a good choice based on the mechanism, efficacy, and tolerability; however, despite adequate dosing, this patient continued to suffer from syncopal episodes; therefore, consideration of adjunctive therapy, such as addition of midodrine, is appropriate. It is important to be vigilant about BP monitoring when adding additional pressor medications, particularly for hypertension, but in this case the benefit outweighed the risks.
